# The Effect of Increasing the Protein Content of Human Milk Fortifier to 1.8 g/100 mL on Growth in Preterm Infants: A Randomised Controlled Trial

**DOI:** 10.3390/nu10050634

**Published:** 2018-05-17

**Authors:** Jessica Reid, Maria Makrides, Andrew J. McPhee, Michael J. Stark, Jacqueline Miller, Carmel T. Collins

**Affiliations:** 1 Healthy Mothers, Babies and Children, South Australian Health and Medical Research Institute, Adelaide, SA 5006, Australia; jessica.reid@adelaide.edu.au (J.R.); maria.makrides@sahmri.com (M.M.); andrew.mcphee@sa.gov.au (A.J.M.); jacqueline.miller@sahmri.com (J.M.); 2 Adelaide Medical School, Discipline of Paediatrics, The University of Adelaide, Adelaide, SA 5006, Australia; 3 Neonatal Medicine, Women’s and Children’s Hospital, Adelaide, SA 5006, Australia; michael.stark@adelaide.edu.au; 4 The Robinson Research Institute, The University of Adelaide, Adelaide, SA 5006, Australia; 5 Nutrition and Dietetics, Flinders University, Adelaide, SA 5006, Australia

**Keywords:** human milk, growth, low birth weight, human milk fortifier

## Abstract

The aim of this study was to assess the effect of feeding high protein human milk fortifier (HMF) on growth in preterm infants. In this single-centre randomised trial, 60 infants born 28–32 weeks’ gestation were randomised to receive a higher protein HMF providing 1.8 g protein (*n* = 31) or standard HMF providing 1 g protein per 100 mL expressed breast milk (EBM) (*n* = 29). The primary outcome was rate of weight gain. Baseline characteristics were similar between groups. There was no difference between high and standard HMF groups for weight gain (mean difference (MD) −14 g/week; 95% CI −32, 4; *p* = 0.12), length gain (MD −0.01 cm/week; 95% CI −0.06, 0.03; *p* = 0.45) or head circumference gain (MD 0.007 cm/week; 95% CI −0.05, 0.06; *p* = 0.79), despite achieving a 0.7 g/kg/day increase in protein intake in the high protein group. Infants in the high protein group had a higher proportion of lean body mass at trial entry; however, there was no group by time effect on lean mass gains over the study. Increasing HMF protein content to 1.8 g per 100 mL EBM does not improve growth in preterm infants born 28–32 weeks’ gestation.

## 1. Introduction

It is well established that fortified human milk improves growth rates in preterm infants [1,2,3]. However, the optimal amount of protein in the fortifier is yet to be determined, partly due to the variability in the protein content of human milk, both within mothers and over time. Too little protein results in a failure to meet protein requirements, estimated to be 4.0–4.5 g/kg/day for infants born <1000 g and 3.5–4.0 g/kg/day for those born 1000–1800 g [4]. Consequently, growth failure in the neonatal period is common in infants fed fortified human milk compared with infants fed preterm formula [5,6,7]. Conversely, too much protein may result in metabolic acidosis [8]. Individualized fortification, based on either the metabolic response of the infant [9,10,11], or the macronutrient content of mother’s milk [12] has been investigated and provides evidence in support of inadequate protein concentration of human milk fortifiers (HMFs) when used in a standardised approach. However, individualised approaches are time consuming and not easily translated to the clinical environment [13]. We previously investigated a fortifier providing 1.4 g compared with 1 g protein per 100 mL human milk in preterm infants <31 weeks’ gestation [14]. While we found no difference in the rate of weight and length gain between groups, there were fewer infants with length <10th percentile at discharge in the high protein group, suggesting a higher protein concentration fortifier may be needed to improve growth. We therefore aimed to determine the effect of further increasing the protein content of HMF to 1.8 g/100 mL compared with 1 g/100 mL, on growth in preterm infants born 28–32 weeks’ gestation.

## 2. Materials and Methods

### 2.1. Study Design

The study was a single centre (Women’s and Children’s Hospital, North Adelaide, South Australia), parallel group randomised controlled trial conducted between February 2012 and May 2013.

### 2.2. Participants

Infants born 28–32 completed weeks’ gestation whose mothers intended to provide breast milk were eligible to participate. Multiple births were eligible and were randomised individually. Infants with a major congenital or chromosomal abnormality likely to affect growth, or where protein therapy was contraindicated (e.g., major heart defects, cystic fibrosis, phenylketonuria, disorders of the urea cycle) were ineligible. Infants likely to transfer to remote locations and infants who had received standard practice HMF for more than four days were also excluded.

### 2.3. Randomisation and Blinding

Infants were randomised to one of two groups: the higher protein intervention group or the standard protein control group. An independent researcher created the randomisation schedule using a computer generated variable block design of 4 and 6. Stratification occurred for sex and gestational age 28–29 weeks and 30–32 weeks. Parents of eligible infants were approached by a neonatologist and followed-up for consent by a research nurse who was not involved in clinical care. Upon consent, infants were randomised by telephoning an independent researcher who held the randomisation schedule and assigned a unique study identification number. Participants, clinicians, outcome assessors and data analysts were blinded to randomisation group.

### 2.4. Interventions

The base HMF used for both trial groups was FM85 Human Milk Supplement (Nestlé Nutrition, Gland, Switzerland) which provides 1.0 g protein and 17.5 kcal when 5 g HMF is added to 100 mL expressed breast milk (EBM). The high protein fortifier was prepared by adding 0.9 g Protifar (Nutricia, Zoetermeer, The Netherlands), a bovine casein-based powder, to the FM 85. This resulted in an additional 0.8 g protein and 3.5 kcal per 100 mL EBM providing 1.8 g protein and 21 kcal when added to 100 mL of EBM. To ensure both fortifiers were isocaloric, thereby eliminating the effect of different energy intakes on growth, 0.9 g Polyjoule (Nutricia, Zoetermeer, The Netherlands), a glucose polymer, was added to the standard fortifier providing an additional 3.5 kcal but no extra protein, giving a total of 1.0 g protein and 21 kcal when added to 100 mL of EBM. The Polyjoule and Protifar supplements were packaged into identical 400-g containers each with a tamper proof seal (Pharmaceutical Packaging Professionals Pty Ltd., Thebarton, Australia). The containers were differentiated by four colour-coded labels to facilitate blinding, with each trial group separately color-coded into two groups. Infant nutrition attendants, under the direction of the Nutrition and Food Services Department, were trained in the preparation of the HMF. Trial fortifier was mixed at the rate of 5 g FM 85 plus either 0.9 g Protifar, or 0.9 g Polyjoule, for the high and standard protein groups respectively, with 4 mL of sterile water, to give a total volume of 8 mL for use with each 100 mL of EBM.

### 2.5. Intervention Administration

The fortifier intervention and control fortifiers were delivered via the enteral tube, immediately prior to a feed (tube, bottle or breast). Trial HMFs were delivered at 8 mL HMF/100 mL EBM with the volume of HMF for each feed ordered daily by the medical or neonatal nurse practitioners. In cases where a mix of EBM and preterm formula was to be given, the trial HMF was only given if EBM was >50% of the total feed. When the infant received a direct breast feed, the timing of administration of the trial product (before, during or after the feed) was at the discretion of the primary care nurse in consultation with the mother. For each day, the trial HMFs were decanted into syringes and labelled with infant identification, volume of HMF and trial details. Syringes were stored refrigerated in the neonatal unit in each infant’s individually labelled container. Any syringes not administered in the 24-h period were recorded and discarded. Fluid balance records were audited daily for compliance with the trial protocol. Administration of trial HMF began as soon as practical after randomisation (within one to two days) and continued until study end, defined as the removal of the naso-gastric tube or estimated date of delivery, whichever came first.

### 2.6. Nutritional Intake

Measured protein and fat content of a weekly sample of unfortified EBM (MilkoScan Minor, Foss, Denmark) were used to represent the weekly composition of EBM [14]. The lactose concentration was assumed to be 6.8 g/100 mL. EBM was only sampled when the supply was surplus to the infant’s requirements. Missing values were substituted with the average macronutrient composition of all available samples (32 of the 45 mothers involved in the study were able to provide breast milk samples). Macronutrient intakes for the study fortifiers, EBM and formula were calculated from the volume ingested, the protein and fat concentration of EBM, and the manufacturer’s information on the study fortifiers and formula. The protein content of the preterm formula in use at the time of the study was 2.2 g/100 mL. Energy content was calculated by using the Atwater factors of 4, 4, and 9 kcal/g for protein, carbohydrate, and fat respectively.

### 2.7. Outcome Assessments

#### 2.7.1. Primary outcome

The primary outcome was rate of weight gain (g/week) from trial start (day of randomisation) to trial end. In addition to routine clinical measurements, a research nurse and J.R. weighed infants on randomisation, weekly and at study end; duplicate weight measurements were taken using electronic balance scales accurate to 5 g. Measurements were repeated if there was a discrepancy ≥10 g, with the average of the two closest measurements used.

#### 2.7.2. Secondary Efficacy and Safety Outcomes

Secondary efficacy outcomes included length and head circumference gain (cm/week), infant weight at study end, small for gestational age (SGA) at study end and body composition (fat-free mass). Length measurements were taken weekly with the infant in the supine position and measured to the nearest 0.1 cm using a recumbent length board. Head circumference was measured weekly using a non-stretching tape placed around the largest occipito-frontal circumference. Duplicate measurements were done and repeated if there was a discrepancy ≥0.5 cm, with the average of the 2 closest measures taken. SGA was defined as below the 10th percentile for infants of the same sex and gestational age, as determined from Australian birth reference data [15]. Fat free (lean) mass was measured weekly by bioelectrical impedance spectroscopy (BIS) using the Imp™ SFB7 (ImpediMed Limited, Queensland, Australia) with the first measurement taken during the first week of the study.

Secondary safety outcomes included feeding tolerance (days feeds interrupted and days to reach enteral intake ≥150 mL/kg/day). A protocol was developed for discontinuation of the trial fortifier based on uraemia (blood urea nitrogen (BUN) concentration >8.0 mmol/L) and/or a metabolic acidosis (base excess <−6 mmol/L) persisting for more than 48 hours. However, no infant met these criteria. Similarly, criteria were defined for the addition of protein to feeds if an infant had poor weight gain defined as <15 g/kg/day over the preceding 7-day period associated with a BUN of <2 mmol/L when feed volumes reached 170 to 180 mL/kg/day. In this case, Protifar could be added at the discretion of the attending neonatologist, in addition to the allocated intervention fortifier. Additional protein was ceased when weight gain of 15 g/kg/day and a BUN >2 mmol/L were achieved.

#### 2.7.3. Biochemical Analyses

Weekly blood samples were taken and BUN, plasma albumin, plasma creatinine, pH and base deficit measured. Blood spots were collected weekly on filter paper and amino acids measured using tandem mass spectrometry (SA Pathology, Neonatal Screening Centre, Adelaide, Australia).

#### 2.7.4. Sample Size and Statistical Analysis

A sample size of 60 (30 per group) would detect a difference in weight gain of 3.31 g per day between the high protein and standard protein groups (80% power, *p* = 0.05). Consultation with the neonatal medical team agreed that this was a clinically important difference on which clinical practice could be changed. Mean weight, length, head circumference and lean mass gains over the trial period, were calculated for each infant using a linear effects model with a random intercept and slope. Using the slope, a linear regression model was fitted for each infant. Clustering (multiple births) was accounted for by using a generalised estimating equation with an independent working correlation matrix. All analyses were intention-to-treat. All models were adjusted for sex and gestational age category (28–29 and 30–32 weeks’ gestation). A per protocol analysis was specified a priori for infants who consumed ≥70% of their prescribed trial fortifier.

#### 2.7.5. Ethics

Ethical approval was granted by the Women’s and Children’s Health Network Human Research Ethics Committee (REC2401/10/14). This trial was registered with the Australian New Zealand Clinical Trials Registry (http://www.anzctr.org.au/) as ACTRN12611001275954.

## 3. Results

### 3.1. Study Population

**Figure 1:**
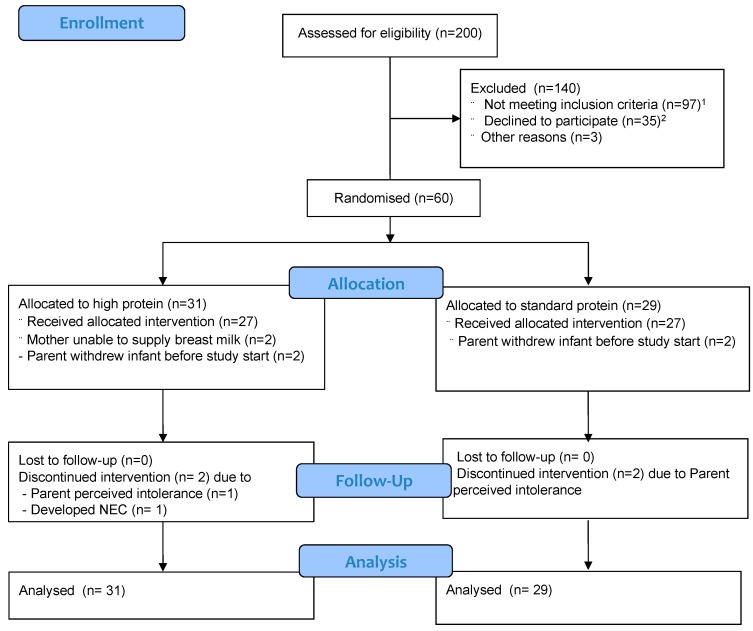
Participant flow through the trial. **^1^** from rural locations (*n* = 52), insufficient milk supply (*n* = 36), required interpreter (*n* = 6); congenital abnormality (*n* = 3); **^2^** did not want to take part (*n* = 25), did not want twins to be randomized individually (*n* = 8), parent not visiting (*n* = 1), immediately transferred to another centre (*n* = 1).

Sixty infants were enrolled in the trial with 31 infants randomised to the high protein group and 29 infants to the standard protein group (Figure 1). There were 31 infants born from multiple births (14 sets of twins, 1 set of triplets). In all multiple births, apart from two sets of twins, the infants were randomly allocated to different interventions. For the triplets, two were randomised to the high protein group and one to the standard protein group. Four infants, two from each group, were withdrawn from the study after randomisation but before the first dose of trial fortifier was administered after parents changed their minds about involvement. A further two infants (twins) in the high protein group did not have any available breast milk and withdrew before the commencement of fortifier. One set of twins and one singleton were withdrawn by the parents midway through the trial due to perceived feeding intolerance and another infant was withdrawn by the clinical team after developing necrotising enterocolitis. In all cases of withdrawal, parents consented to the ongoing collection of data and all were included in intention-to-treat analyses. Baseline infant and maternal demographic, clinical and nutritional characteristics at randomisation were comparable between groups except that there were more male infants in the high protein group, *n* = 16 (52%) than the standard protein group, *n* = 12 (41%), the mean ± SD birth weight was lower in the higher protein group (1483 ± 423 g versus 1551 ± 407 g in the high and standard groups, respectively) and there were more infants classified as SGA for weight in the high protein group, *n* = 5 (16%) than the standard protein group, *n* = 1 (3%) (Table 1).

### 3.2. Nutritional Management

Forty infants received standard ward HMF, S-26 SMA HMF (Wyeth Nutrition) while waiting for consent, 18 in the high and 22 in the standard protein group (Table 1). The remaining twenty trial infants started immediately on their allocated trial intervention.

Nutritional intake of the infants for the first 28 days of the study did not differ between the groups except that the high protein group received more protein (mean ± SD 4.2 ± 1.3 vs. 3.5 ± 0.93 g/kg/day in the high and standard protein groups respectively). The protein concentration of the EBM was not different between groups (mean ± SD 1.43 ± 0.27 and 1.45 ± 0.28 g protein/100 mL in the high and standard groups, respectively) and the difference in protein intake was due to more protein derived from the HMF (mean ± SD 1.9 ± 1.2 and 1.2 ± 0.6 g/kg/day, in the high and standard groups, respectively. Energy intakes and fluid volume were similar between the groups (energy: mean ± SD 124 ± 34 and 126 ± 27 kcal/kg/day and fluid: mean ± SD 154 ± 39 and 157 ± 32 mL/kg/day in the high and standard groups, respectively). The high protein group received 83% (±32) of their total enteral intake as EBM compared with the control group who received 90% (±23).

### 3.3. Primary Outcome

There was no difference in the rate of weight gain between groups (Table 2) (mean (95% CI) high protein 245 (230, 260) g/week and standard protein 258 (244, 272) g/week, adjusted mean difference −14 (−32, 4) *p* = 0.12). Results were similar when analysed per protocol (Table 2).

### 3.4. Secondary Outcomes

#### 3.4.1. Growth

There were no differences in rate of length or head circumference gain (Table 2). High protein HMF infants weighed less at study end but this was not statistically significant (Table 2) and is consistent with the difference in birth weight between the groups (Table 1). There were no differences in length or head circumference at study end between the groups (Table 2). There was no difference in SGA status for weight between high and standard protein HMF groups at the end of the study (*n* = 8, 25%, and *n* = 3, 10% SGA infants in the high and standard protein groups, respectively, adjusted Relative Risk (95% CI); 2.5 (0.8, 7.9), *p* = 0.11).

**Figure 2:**
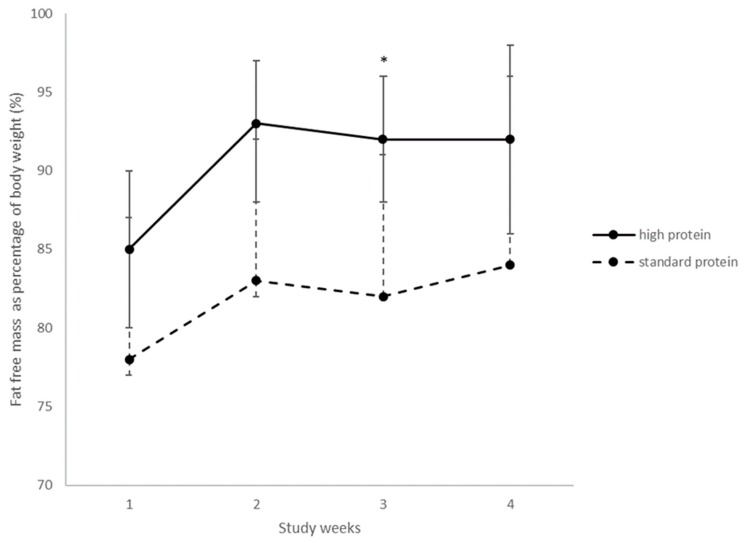
Fat free mass as a proportion of body weight for the first four weeks of the trial. Values are means, error bars are 95% CI. High protein *n* = 30, 30, 27, 26 and standard protein 29, 27, 26, 23 in weeks 1, 2, 3, 4 respectively. Adjusted for sex and gestational age, group interaction, *p* = 0.03, time interaction, *p* = 0.01. group × time interaction *p* = 0.84; * *p* = 0.04.

Over the first four weeks of the trial, when >75% of participants were still in hospital, fat free (lean) mass was measured with the week one measurement taken a mean of 8 ± SD 2 days after randomisation. Fat free mass as a proportion of body weight (Figure 2) from weeks one to four was greater in high protein group infants than standard protein group infants (*p* = 0.03). However, there was no significant group by time interaction (*p* = 0.84). At week three alone, there was a significant increase for fat free mass as a proportion of body weight in the high protein group (*p* = 0.04).

#### 3.4.2. Biochemistry

Due to the variable nature of blood chemistry data and length of hospital stay (to discharge), only the first three trial weeks could be accurately analysed using a linear mixed effects model.

**Figure 3:**
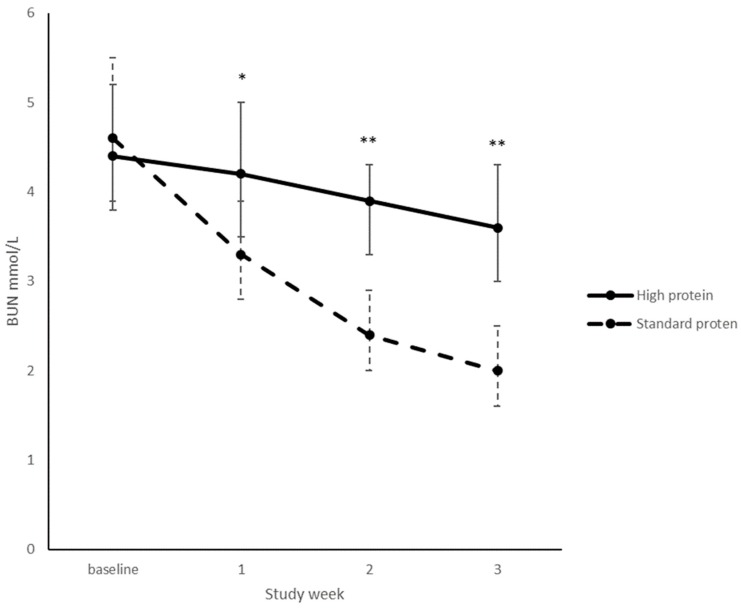
BUN from randomisation to week 3. Values are mean, error bars are 95% CI. High protein: *n* = 31, 28, 26, 25; Standard protein: *n* = 29, 26, 24, 24 for weeks baseline, 1, 2, 3. Adjusted for sex and GA, overall group effect <0.001, group * week interaction, *p* <0.001, * *p* = 0.04; ** *p* <0.001.

There was a significant group by time interaction for BUN levels (*p* < 0.001) with BUN levels significantly increased in the high protein group (Figure 3). This difference continued for the duration of the trial (*p* < 0.001). There were 12 occurrences in nine separate infants where BUN levels were measured over the pre-specified safety threshold of 8 mmol/L. Seven of these occurred during baseline blood tests taken at randomisation and were therefore not a result of the intervention. Six of these infants had BUN measurements in the normal range at their next weekly blood test. One infant had a BUN measurement >8 mmol/L at week one; the infant did not have another BUN measurement over 8 mmol/L for the rest of the trial. Two other infants, both in the high protein group, recorded BUN concentrations >8.0 mmol/L, peaking at 8.8 mmol/L, on five occasions, however the base excess remained above −6 mmol/L with no other abnormal biochemistry. There was one occurrence of an infant in the standard protein group requiring additional protein due to poor weight gain and BUN <2 mmol/L.

There were no group by time interactions or group differences for albumin, creatinine, glucose, pH (results not shown). Phenylalanine (Phe) and tyrosine (Tyr), amino acids associated with increased protein intake, were both increased in the high protein group compared to the standard group at study week 3 (Phe median (IQR) μmol/L: 33 (28–42) vs. 25 (23–30), *p* <0.001 and Tyr median (IQR) μmol/L: 196 (151–267) vs. 128 (99–172) μmol/L, *p* <0.003 in the high and standard groups respectively.

#### 3.4.3. Clinical Outcomes

High protein HMF infants were significantly more likely to have feeds interrupted (11 (35%) vs. 6 (21%), *p* = 0.01, in the high and standard protein groups, respectively) Table 3. There was no significant difference in the number of days spent on parenteral nutrition, days of intravenous lipid or the days taken to reach full enteral feeds. Likewise, there was no significant difference between the groups for any other clinical outcome (Table 3).

## 4. Discussion

The aim of this study was to assess the effect of a higher protein HMF on preterm infant growth. Our trial interventions resulted in the high protein group infants receiving 0.7 g/kg/day more protein than infants in the standard protein group, with mean protein intakes within recommended ranges for both groups. Despite this, there were no differences in growth between the two groups. The accumulation of fat free mass and fat mass, also did not differ between groups. While the higher protein group had a greater proportion of fat free mass from week one, the absence of a baseline measurement makes the interpretation of this difficult. It is unlikely that the intervention would have had an effect in the first week of the study, particularly as the change in fat free mass over time did not differ between groups. A significant difference between groups was noted at week three only and the implication of this is unclear. It is possible that this is a chance finding of no clinical significance.

These results are confirmed by a recent study by Maas et al. [16] who compared 1 and 1.8 g protein concentration in powdered HMFs in a similar population to ours and found no difference in growth. Their trial interventions achieved a 0.6 g/kg/day median greater intake of protein, similar to our study, and protein intakes were within recommendations. Growth rates in both studies approximated foetal growth rates. A further two studies compared two different, newly formulated liquid HMFs with higher protein concentrations, with standard powdered HMFs. Moya et al. [17] compared Mead Johnson Nutrition products: a liquid fortifier with an Enfamil powdered fortifier, which when mixed with EBM provided 3.2 and 2.6 g protein/100 mL, respectively, equating to an additional 1.8 and 1.1 g protein. Kim et al. [18], in a non-inferiority trial, compared the Abbott Nutrition products of Similac HMF liquid, providing 3.6 g protein/100 kcal when mixed with EBM, with Similac HMF powder providing 3 g protein/100 kcal when mixed. These comparisons equate to an additional 1.6 and 1 g protein added to 100 mL EBM in the liquid and powder, respectively. The populations were similar between studies [17,18] except that Moya et al. [17] inclusion criteria (≤30 weeks’ gestation, birth weight ≤1250 g) resulted in a slightly less mature and smaller population than in both Kim et al. [18] study and this current study. Neither study [17,18] showed a difference in weight gain between groups, however, Moya et al. [17] found improved length gain with the higher protein. Both studies found infants in the high protein group were heavier at study end. Almost half the participants in Moya’s study were <1000 g at birth; hence their protein requirements of 4 to 4.5 g/kg would have been met by the high, but not the control, protein fortifier at volumes of 150 mL/kg. This may explain the effect seen on length gain. Two other studies have compared fortifiers containing 1 and 1.4 g protein added to 100 mL EBM with mixed results. Our previous trial [14] showed no effect of increased protein on growth, although did show a reduction in the number of infants SGA for length at discharge. However, Rigo et al. [19], in a non-inferiority trial, found improved weight gain of 2.3 g/day with the higher protein fortifier. The trial products in both these studies were similar, as were the population. It is possible that the smallest infants, with the highest protein needs, are the ones to benefit most from increased protein and that the larger sample size in Rigo (*n* = 153) compared to that in Miller (*n* = 92) elucidated the differences. Taken collectively, these results and ours suggest that protein concentrations in HMFs of 1.8 g provide no additional benefit in the population studied, but smaller infants are worthy of further investigation.

The significantly elevated BUN levels seen at weeks 1, 2 and 3 were expected and have occurred in other high protein nutritional intervention studies [9,14,17]. Assuming adequate renal function, BUN is proportional to protein intake [20] and is often used as a crude marker of protein sufficiency. Low BUN levels suggest inadequate protein intake and high levels indicate possible excessive intake [9]. Blood phenylalanine and tyrosine concentrations were also significantly increased in the higher protein group, in week 3 only, and this is unlikely to be clinically significant. There were no differences in creatinine, albumin or other biochemical markers suggesting the intervention did not harm the infants.

A strength of this study is the rigour with which dietary intake and growth were assessed. The protein and fat concentrations of EBM were measured, rather than assumed, resulting in accurate reporting of dietary intake and confirmation that, despite the variability of protein in EBM, we achieved a mean intake difference of 0.7 g/kg/day of protein between groups. Similarly, we measured both growth and body composition in an attempt to discern differences in weight gain arising from extra protein. This trial also has some limitations. Although all infants were included in the analyses, there were 10 who either did not receive, or ceased the intervention, which may have impacted results. In addition, the pragmatic nature of this trial may have influenced results as clinicians may have adjusted feed regimes if poor weight gain was identified. There was one instance of extra Protifar prescribed to an infant in the standard protein group and subtle increases in feed volume may also have occurred although volume of intake was not different between groups. This may have made it more difficult to detect differences between intervention groups. We used BIS to determine fat and fat free mass. BIS is the only cot-side technique available where infants requiring respiratory support can be assessed. While accuracy of BIS at the individual level is poor, BIS provides a useful means of determining differences in body composition between population means [21].

Many of the recent trials discussed have already achieved mean growth rates approaching intra-uterine growth, with similar growth rates between groups. Findings from this current study are only generalisable to a similar population (infants born 28–32 week’s gestation). Therefore, to explicate the subtle effects of increasing protein on growth, future trials may need to focus on birth weight categories as they relate to protein requirements (i.e., <1000 g and 1000–1800 g). Due to the small proportion of infants born <1000 g, large multi-centre trials will be needed to tease out the effect.

## 5. Conclusions

Increasing the protein concentration of HMF from 1.0 to 1.8 g protein added per 100 mL EBM does not improve growth in preterm infants born 28–32 weeks’ gestation.

## Figures and Tables

**Table 1:** Baseline infant and maternal characteristics.

Characteristic	High Protein (*n* = 31)	Standard Protein (*n* = 29)
**Infant characteristics**		
Singleton	15 (48)	16 (55)
Twin	15 (48)	12 (41)
Triplet	2 (7)	1 (3)
Gestational age (week)	30.5 ± 1.5	30.1 ± 1.4
28–29 weeks’ gestation	10 (32)	9 (31)
30–32 weeks’ gestation	21 (68)	20 (69)
Male infants	16 (52)	12 (41)
Birth weight (g)	1483 ± 423	1551 ± 407
SGA for weight at birth	5 (16)	1 (3)
Birth length (cm)	40.0 ± 3.3	40.2 ± 2.8
Head circumference (cm)	28.5 ± 3	28.5 ± 1.8
Infants received standard ward HMF before randomisation	18 (58)	22 (76)
Length of standard ward fortification before trial HMF start (day)	1.3 ± 1.7	2.0 ± 1.5
Time between birth and trial HMF start (day)	8.9 ± 3.2	9.0 ± 2.5
**Maternal characteristics**		
Maternal age (years)	29.9 ± 6.3	31.7 ± 5.3
Mother smoked during pregnancy	5 (16.1)	3 (10.3)
Caucasian	27 (96)	23 (82)
Primiparous	19 (61.3)	12 (41.4)
Previous preterm birth	4 (33.3)	6 (35.3)

Data are presented as *n* (%) or mean ± SD.

**Table 2:** Anthropometric changes over the study.

	Intention to Treat Analyses				Per Protocol Analyses ^1^			
	High Protein (*n* = 31)	Standard Protein (*n* = 29)	Adjusted Mean Difference ^2^	*p* ^2^	High Protein (*n* = 21)	Standard Protein (*n* = 23)	Adjusted Mean Difference ^2^	*p* ^2^
Weight gain (g/week)	245 (230, 260)	258 (244, 272)	−14 (−32, 4)	0.12	245 (228, 262)	262 (247, 277)	−15 (−36, 5)	0.14
Length gain (cm/week)	1.1 (1.1, 1.2)	1.1 (1.1, 1.2)	−0.01 (−0.06, 0.03)	0.45	1.1 (1.1, 1.2)	1.2 (1.1, 1.2)	−0.01 (−0.06, 0.04)	0.62
Head circumference gain (cm/week)	1.1 (1.0, 1.1)	1.1 (1.0,1.1)	0.007 (−0.05, 0.06)	0.79	1.1 (1.1, 1.1)	1.1 (1.1, 1.1)	−0.004 (−0.06, 0.05)	0.88
Weight at study end (g) ^3^	2658 (2544, 2771)	2757 (2632, 2883)	−100 (−251, 50)	0.19	2646 (2489, 2805)	2815 (2675, 2955)	−157 (−341, 28)	0.1
Length at study end (cm)	45.2 (44.5, 45.9)	45.8 (45.0, 46.6)	−0.5 (−1.3, 0.3)	0.19	45.2 (44.4, 46.0)	46.3 (45.6, 47)	−0.86 (−1.85, 0.12)	0.09
Head circumference at study end (cm)	33.1 (32.5, 33.6)	33.0 (32.4, 33.7)	0.03 (−0.6, 0.7)	0.92	33.3 (32.7, 33.9)	33.6 (33.0, 34.1)	−0.16 (−0.90, 0.57)	0.66

Data are presented as mean, (95% CI); ^1^ For inclusion in ‘per protocol’ analysis, infants must have consumed 70% or more of their trial group HMF; ^2^ adjusted for sex and gestational age; ^3^ study end defined as removal of naso-gastric tube or term equivalent, whichever came first.

**Table 3:** Feeding and clinical management.

Variable	High Protein (*n* = 31)	Standard Protein (*n* = 29)	*p*
Infant required enteral protein supplementation ^1^	0	1 (3.4)	0.48
Feeding interrupted ^2^	11 (35)	6 (21)	0.01
Days receiving parenteral nutrition	10 (7, 13)	9 (7, 11)	0.34
Days of intravenous lipid	4 (3, 7)	4 (3, 6)	0.72
Days to full enteral feeds ^3^	8 (6, 10)	8 (7, 10)	0.72
Confirmed necrotizing enterocolitis	1 (3.2)	0	>0.99
Oxygen at discharge	2 (6.5)	1 (3.4)	0.15
Late onset sepsis	1 (3.2)	0	>0.99

Data are reported as *n* (%) or mean (95% CI).^1^ One infant in the standard protein group was prescribed a protein supplement (Protifar) ^2^ Feeding interrupted was defined as one of more feeds not given in a day; ^3^ Full enteral feeds was defined as 150 mL/kg/day).
